# Supplementary material for: Plectronoceratids (Cephalopoda) from the latest Cambrian at Black Mountain, Queensland, reveal complex three-dimensional siphuncle morphology, with major taxonomic implications
Source: PeerJ. 2024 Feb 29;12:e17003. doi: 10.7717/peerj.17003 (PMC10909373; doi:10.7717/peerj.17003)
Supplement: Supplemental Information 4 — In the linear regression models, species are included as interaction terms. Significant p-values (< 0.05) are highlighted in bold. Non-significant p-values cannot reject that the slopes of the ontogenetic trajectories are statistically distinct (i.e., parallel). [file peerj-12-17003-s004.pdf]

Table S1. P-values of pairwise comparisons (ANOVA) of regression coefficients of relative cameral length (=RCL) between species. In the linear regression models, species are included as interaction terms. Significant p-values (< 0.05) are highlighted in bold. Non-significant p-values cannot reject that the slopes of the ontogenetic trajectories are statistically distinct (i.e., parallel).

**RCL**	*Pa. mutabile*	*Pl. cambria*	*S. bullatum*	*S. endogastrum*	*S. inflatum*	*S. magicum*	*S. marywadeae*	*S. shanxiense*	*S. sibiriense*	*S. sinense*	*S. wanwanense*
Pa. mutabile		0.5649	**< 0.0001**	0.3869	**0.0148**	0.1217	**< 0.0001**	0.0132	0.0857	**0.0001**	**< 0.0001**
Pl. cambria	0.5649		**0.0344**	0.6689	0.6241	0.1391	**0.0166**	0.4440	0.0600	**0.0484**	0.0604
S. bullatum	**< 0.0001**	**0.0344**		0.1314	**0.0039**	0.6789	0.1365	**0.0005**	0.8266	0.2464	0.0771
S. endogastrum	0.3869	0.6689	0.1314		0.8011	0.2769	0.1235	0.7557	0.1324	0.1583	0.2172
S. inflatum	**0.0148**	0.6241	**0.0039**	0.8011		0.8549	**< 0.0001**	0.6424	0.7268	0.2514	**0.0016**
S. magicum	0.1217	0.1391	0.6789	0.2769	0.8549		0.7539	0.4689	0.4728	0.8423	0.8314
S. marywadeae	**< 0.0001**	**0.0166**	0.1365	0.1235	**< 0.0001**	0.7539		**< 0.0001**	0.7676	0.4559	0.2617
S. shanxiense	0.0132	0.4440	**0.0005**	0.7557	0.6424	0.4689	**< 0.0001**		0.2538	**0.0137**	**0.0006**
S. sibiriense	0.0857	0.0600	0.8266	0.1324	0.7268	0.4728	0.7676	0.2538		0.6089	0.7664
S. sinense	**0.0001**	**0.0484**	0.2464	0.1583	0.2514	0.8423	0.4559	**0.0137**	0.6089		0.7153
S. wanwanense	**< 0.0001**	0.0604	0.0771	0.2172	**0.0016**	0.8314	0.2617	**0.0006**	0.7664	0.7153	
