# Supplementary material for: Plectronoceratids (Cephalopoda) from the latest Cambrian at Black Mountain, Queensland, reveal complex three-dimensional siphuncle morphology, with major taxonomic implications
Source: PeerJ. 2024 Feb 29;12:e17003. doi: 10.7717/peerj.17003 (PMC10909373; doi:10.7717/peerj.17003)
Supplement: Supplemental Information 5 — In the linear regression models, species are included as interaction terms. Significant p-values (< 0.05) are highlighted in bold. Non-significant p-values cannot reject that the slopes of the ontogenetic trajectories are statistically distinct (i.e., parallel). [file peerj-12-17003-s005.pdf]

Table S2. P-values of pairwise comparisons (ANOVA) of regression coefficients of height expansion rate (= ER_h) between species. In the linear regression models, species are included as interaction terms. Significant p-values (< 0.05) are highlighted in bold. Non-significant p-values cannot reject that the slopes of the ontogenetic trajectories are statistically distinct (i.e., parallel).

**Er_h**	*Pa. mutabile*	*Pl. cambria*	*S. bullatum*	*S. endogastrum*	*S. inflatum*	*S. magicum*	*S. marywadeae*	*S. shanxiense*	*S. sibiriense*	*S. sinense*	*S. wanwanense*
Pa. mutabile		0.1119	0.7736	0.7750	0.4950	0.8954	0.5441	**0.0060**	0.4165	0.0596	0.7557
Pl. cambria	0.1119		0.6165	0.3660	0.4521	0.1645	0.5441	0.9255	**0.0120**	0.0433	0.7302
S. bullatum	0.7736	0.6165		0.9681	**0.0332**	0.9848	0.2838	0.0357	0.7480	0.2111	0.6623
S. endogastrum	0.7750	0.3660	0.9681		0.8174	0.8356	0.9918	0.5298	0.2140	0.3304	0.9972
S. inflatum	0.4950	0.4521	**0.0332**	0.8174		0.8456	**< 0.0001**	**0.0043**	0.9519	0.8340	**0.0163**
S. magicum	0.8954	0.1645	0.9848	0.8356	0.8456		0.9266	0.4444	0.0532	0.3220	0.9674
S. marywadeae	0.5441	0.5441	0.2838	0.9918	**< 0.0001**	0.9266		**0.0074**	0.6627	0.0500	0.7296
S. shanxiense	**0.0060**	0.9255	0.0357	0.5298	**0.0043**	0.4444	**0.0074**		0.1652	**0.0025**	0.1199
S. sibiriense	0.4165	**0.0120**	0.7480	0.2140	0.9519	0.0532	0.6627	0.1652		0.9542	0.8095
S. sinense	0.0596	0.0433	0.2111	0.3304	0.8340	0.3220	0.0500	**0.0025**	0.9542		0.2943
S. wanwanense	0.7557	0.7302	0.6623	0.9972	**0.0163**	0.9674	0.7296	0.1199	0.8095	0.2943	
