# Supplementary material for: Plectronoceratids (Cephalopoda) from the latest Cambrian at Black Mountain, Queensland, reveal complex three-dimensional siphuncle morphology, with major taxonomic implications
Source: PeerJ. 2024 Feb 29;12:e17003. doi: 10.7717/peerj.17003 (PMC10909373; doi:10.7717/peerj.17003)
Supplement: Supplemental Information 6 — In the linear regression models, species are included as interaction terms. Significant p-values (< 0.05) are highlighted in bold, representing cases where the trajectories may be parallel, but the intercept is significantly different, i.e., RCL is distinct at the same conch height. [file peerj-12-17003-s006.pdf]

Table S3. P-values of pairwise comparisons (ANOVA) of intercepts of relative cameral length (=RCL) between species, where no significant difference in slope was detected (Table S1). In the linear regression models, species are included as interaction terms. Significant p-values (< 0.05) are highlighted in bold, representing cases where the trajectories may be parallel, but the intercept is significantly different, i.e., RCL is distinct at the same conch height.

**RCL**	*Pa. mutabile*	*Pl. cambria*	*S. bullatum*	*S. endogastrum*	*S. inflatum*	*S. magicum*	*S. marywadeae*	*S. shanxiense*	*S. sibiriense*	*S. sinense*	*S. wanwanense*
Pa. mutabile		**< 0.0001**		**< 0.0001**		**< 0.0001**		**< 0.0001**	**< 0.0001**		
Pl. cambria	**< 0.0001**			**0.0002**	0.0828	**0.0005**		**< 0.0001**	**0.0045**		**0.0001**
S. bullatum				**< 0.0001**		**< 0.0001**	**< 0.0001**		**< 0.0001**	**< 0.0001**	**< 0.0001**
S. endogastrum	**< 0.0001**	**0.0002**	**< 0.0001**			**0.0036**	**0.0016**	**0.0263**	**0.0015**	**0.0051**	**0.0106**
S. inflatum		0.0828				**< 0.0001**	**< 0.0001**		**< 0.0001**	**< 0.0001**	**< 0.0001**
S. magicum	**< 0.0001**	**0.0005**	**< 0.0001**	**0.0036**	**< 0.0001**		0.1559	0.2656	**0.0338**	0.1105	0.2366
S. marywadeae			**< 0.0001**	**0.0016**	**< 0.0001**	0.1559			**< 0.0001**	**< 0.0001**	**< 0.0001**
S. shanxiense	**< 0.0001**	**< 0.0001**		**0.0263**		0.2656			**< 0.0001**		
S. sibiriense	**< 0.0001**	**0.0045**	**< 0.0001**	**0.0015**	**< 0.0001**	**0.0338**	**< 0.0001**	**< 0.0001**		0.8176	0.8740
S. sinense			**< 0.0001**	**0.0051**	**< 0.0001**	0.1105	**< 0.0001**		0.8176		**0.0022**
S. wanwanense		**0.0001**	**< 0.0001**	**0.0106**	**< 0.0001**	0.2366	**< 0.0001**		0.8740	**0.0022**	
