# Supplementary material for: Plectronoceratids (Cephalopoda) from the latest Cambrian at Black Mountain, Queensland, reveal complex three-dimensional siphuncle morphology, with major taxonomic implications
Source: PeerJ. 2024 Feb 29;12:e17003. doi: 10.7717/peerj.17003 (PMC10909373; doi:10.7717/peerj.17003)
Supplement: Supplemental Information 7 — In the linear regression models, species are included as interaction terms. Significant p-values (< 0.05) are highlighted in bold, representing cases where the trajectories may be parallel, but the intercept is significantly different, i.e., ERh is distinct at the same conch height. [file peerj-12-17003-s007.pdf]

Table S4. P-values of pairwise comparisons (ANOVA) of intercepts of height expansion rate (= ER_h) between species, where no significant difference in slope was detected (Table S2). In the linear regression models, species are included as interaction terms. Significant p-values (< 0.05) are highlighted in bold, representing cases where the trajectories may be parallel, but the intercept is significantly different, i.e., ER_h is distinct at the same conch height.

**Er_h**	*Pa. mutabile*	*Pl. cambria*	*S. bullatum*	*S. endogastrum*	*S. inflatum*	*S. magicum*	*S. marywadeae*	*S. shanxiense*	*S. sibiriense*	*S. sinense*	*S. wanwanense*
Pa. mutabile		0.1136	0.4909	0.0865	0.5581	0.0779	0.4390	0.2536	**0.0445**	0.1009	0.6490
Pl. cambria	0.1136		0.1757	**0.0042**	0.1859	**0.0036**	**0.0499**	0.0576		**0.0075**	0.2855
S. bullatum	0.4909	0.1757		**0.0012**		**0.0019**	**< 0.0001**	**0.0012**	**0.0004**	**0.0012**	**0.0047**
S. endogastrum	0.0865	**0.0042**	**0.0012**			0.2897	0.7692	0.6873	0.2107	0.4442	0.8692
S. inflatum	0.5581	0.1859				**0.0346**	**0.0385**			**0.0268**	**0.0349**
S. magicum	0.0779	**0.0036**	**0.0019**	0.2897	**0.0346**		0.8373	0.7852	0.0984	0.5358	0.9094
S. marywadeae	0.4390	**0.0499**	**< 0.0001**	0.7692	**0.0385**	0.8373			**< 0.0001**	**< 0.0001**	**< 0.0001**
S. shanxiense	0.2536	0.0576	**0.0012**	0.6873		0.7852			**< 0.0001**		**0.0003**
S. sibiriense	**0.0445**		**0.0004**	0.2107		0.0984	**< 0.0001**	**< 0.0001**		0.4047	0.8939
S. sinense	0.1009	**0.0075**	**0.0012**	0.4442	**0.0268**	0.5358	**< 0.0001**		0.4047		0.6957
S. wanwanense	0.6490	0.2855	**0.0047**	0.8692	**0.0349**	0.9094	**< 0.0001**	**0.0003**	0.8939	0.6957	
